# Supplementary material for: An Analysis of Transcriptomic Burden Identifies Biological Progression Roadmaps for Hematological Malignancies and Solid Tumors
Source: Biomedicines. 2022 Oct 27;10(11):2720. doi: 10.3390/biomedicines10112720 (PMC9687799; doi:10.3390/biomedicines10112720)
Supplement: Supplementary file 1 [file biomedicines-10-02720-s001.zip › R code for Bioprogression.pdf]

```
### 06/09/2021### modded 06/08/2021

getLCPM <- function(x,glen,z) {

 # x: un-normalized counts matrix or TMM normalized lcpm matrix
 # glen: ensembl gene ID to gene symbol
 # z: x normalized or not (TRUE/FALSE)
 # x <- readRDS("NCICCR-DLBCL_htseq_counts.rds")
 # glen <- readRDS("ensembl_ext_gene_name_mean_tx_length.rds")
 # lcpm <- getLCPM(x,glen,FALSE)

 if(length(grep("ENSG", rownames(x))) == nrow(x)){

        x <- data.frame(GeneID=rownames(x), x, stringsAsFactors=F)

        if(length(grep(".",x$GeneID)) > 0){

                for(i in 1:nrow(x)){x$GeneID[i] <- strsplit(x$GeneID[i],"\\.")[[1]][1]}

        }

        x <- merge(glen, x, by="GeneID")
        rownames(x) <- x$Gene

        if(!z){

                y <- DGEList(counts=x[, -c(1:4)], genes= x[, 1:4])
                keep <- filterByExpr(y)
                y <- y[keep, , keep.lib.sizes=FALSE]
                y <- calcNormFactors(y, method = "TMM")
                lcpm <- cpm(y, log=TRUE)
                return(lcpm)

        }else{

                return(as.matrix(x[, -c(1:4)]))

        }

 }else{

        cat("Please use ensembl gene ID starting with ENSG! \n")
        return(NULL)
 }

}

getMS <- function(x) {
```

```
 y <- x

 mm <- data.frame(Rmin=rep(0, nrow(x)), Rmax=rep(0, nrow(x)))

 for(i in 1:nrow(x)){

        mm$Rmin[i] <- min(x[i, ])
        mm$Rmax[i] <- max(x[i, ])

 }

 for(i in 1:nrow(x)){

        for(j in 1:ncol(x)){

                y[i,j] <- (x[i,j] - mm$Rmin[i])/(mm$Rmax[i] - mm$Rmin[i])

        }
 }

 rm(mm)

 return(y)

}

getGB_PT <- function(data) {

 setGB <- 0

 for(i in 1:nrow(data)){

        setGB[i] <- (median(data[i, ]) - min(data[i, ]))/(max(data[i, ]) - min(data[i, ]))

 }

 names(setGB) <- rownames(data)
 setGB <- setGB[order(setGB)]

 setPT <- matrix(rep(0, 2*ncol(data)), nrow=2, ncol=ncol(data), byrow = TRUE)
 colnames(setPT) <- colnames(data)
 rownames(setPT) <- c("sum","PT")

 setPT[1,] <- colSums(data)

 for(i in 1:ncol(data)){
```

```
        setPT[2,i] <- (setPT[1,i] - min(setPT[1,]))/(max(setPT[1,]) - min(setPT[1,]))

}

setPTonly <- setPT[2,]
setPTonly <- setPTonly[order(setPTonly)]

data <- data[names(setGB), ]
data <- data[, names(setPTonly)]

gsData <- getMS(data)

setPT <- data.frame(sample=names(setPTonly), PT=setPTonly, PTlabel="", stringsAsFactors=F)

setPT[setPT$PT <= 0.25, "PTlabel"]  <- "EarlyStage"
setPT[setPT$PT >= 0.4 & setPT$PT <= 0.6,  "PTlabel"]  <- "MidStage"
setPT[setPT$PT >= 0.75,  "PTlabel"]  <- "LateStage"

rn1 <- setPT[setPT$PT <= 0.25, ]
rn2 <- setPT[setPT$PT >= 0.4 & setPT$PT <= 0.6, ]
rn3 <- setPT[setPT$PT >= 0.75, ]

sel <- c(nrow(rn1),nrow(rn2),nrow(rn3))
sel <- sel[order(sel)][2]

if(length(grep("EarlyStage",setPT$PTlabel)) > sel){
        setPT$PTlabel[(sel + 1):max(grep("EarlyStage",setPT$PTlabel))] <- ""
}else if(length(grep("EarlyStage",setPT$PTlabel)) < sel){

        if(min(grep("MidStage",setPT$PTlabel)) > sel){
                setPT$PTlabel[1:sel] <- "EarlyStage"
        }else{
                setPT$PTlabel[1:(min(grep("MidStage",setPT$PTlabel)) - 1)] <- "EarlyStage"
        }

}

if(length(grep("LateStage",setPT$PTlabel)) > sel){
        setPT$PTlabel[min(grep("LateStage",setPT$PTlabel)):(max(grep("LateStage",setPT$PTlabel)) - sel) ]
<- ""
}else if(length(grep("LateStage",setPT$PTlabel)) < sel){

        if((max(grep("LateStage",setPT$PTlabel)) - max(grep("MidStage",setPT$PTlabel))) > sel){
                setPT$PTlabel[(max(grep("LateStage",setPT$PTlabel)) - sel +
1):max(grep("LateStage",setPT$PTlabel))] <- "LateStage"
        }else{
                setPT$PTlabel[(max(grep("MidStage",setPT$PTlabel)) +
1):max(grep("LateStage",setPT$PTlabel))] <- "LateStage"
```

```
        }

}

if(length(grep("MidStage",setPT$PTlabel)) > sel){

        if(sel %% 2 == 0){

        setPT$PTlabel[c(min(grep("MidStage",setPT$PTlabel)):(round(median(grep("MidStage",setPT$PTlabel)),0) - round(sel/2,0)), (round(median(grep("MidStage",setPT$PTlabel)),0) + round(sel/2,0) + 1):max(grep("MidStage",setPT$PTlabel)))] <- ""
        }else{

        setPT$PTlabel[c(min(grep("MidStage",setPT$PTlabel)):(round(median(grep("MidStage",setPT$PTlabel)),0) - round(sel/2,0) - 1), (round(median(grep("MidStage",setPT$PTlabel)),0) + round(sel/2,0) + 1):max(grep("MidStage",setPT$PTlabel)))] <- ""
        }

}else if(length(grep("MidStage",setPT$PTlabel)) < sel){

        if((min(grep("LateStage",setPT$PTlabel)) - max(grep("EarlyStage",setPT$PTlabel))) > sel){

                if((median(grep("MidStage",setPT$PTlabel)) - round(sel/2,0)) >= max(grep("EarlyStage",setPT$PTlabel)) & (median(grep("MidStage",setPT$PTlabel)) + round(sel/2,0)) <= min(grep("LateStage",setPT$PTlabel))){

                        setPT$PTlabel[(median(grep("MidStage",setPT$PTlabel)) - round(sel/2,0) + 1):(median(grep("MidStage",setPT$PTlabel)) + round(sel/2,0))] <- "MidStage"

                }else if((median(grep("MidStage",setPT$PTlabel)) - round(sel/2,0)) >= max(grep("EarlyStage",setPT$PTlabel)) & (median(grep("MidStage",setPT$PTlabel)) + round(sel/2,0)) > min(grep("LateStage",setPT$PTlabel))){

                        setPT$PTlabel[(median(grep("MidStage",setPT$PTlabel)) - round(sel/2,0) + 1):(min(grep("LateStage",setPT$PTlabel)) - 1)] <- "MidStage"

                }else if((median(grep("MidStage",setPT$PTlabel)) - round(sel/2,0)) < max(grep("EarlyStage",setPT$PTlabel)) & (median(grep("MidStage",setPT$PTlabel)) + round(sel/2,0)) <= min(grep("LateStage",setPT$PTlabel))){

                        setPT$PTlabel[(max(grep("EarlyStage",setPT$PTlabel)) + 1):(median(grep("MidStage",setPT$PTlabel)) + round(sel/2,0))] <- "MidStage"

                }else if((median(grep("MidStage",setPT$PTlabel)) - round(sel/2,0)) < max(grep("EarlyStage",setPT$PTlabel)) & (median(grep("MidStage",setPT$PTlabel)) + round(sel/2,0)) > min(grep("LateStage",setPT$PTlabel))){
```

```
                            setPT$PTlabel[(max(grep("EarlyStage",setPT$PTlabel)) +
 1):(min(grep("LateStage",setPT$PTlabel)) - 1)] <- "MidStage"

                  }

        }else{
                  setPT$PTlabel[(max(grep("EarlyStage",setPT$PTlabel)) +
 1):(min(grep("LateStage",setPT$PTlabel)) - 1)] <- "MidStage"
        }

 }

 rm(rn1)
 rm(rn2)
 rm(rn3)

 if(min(grep("MidStage",setPT$PTlabel)) - max(grep("EarlyStage",setPT$PTlabel)) > 1){
        setPT$PTlabel[(max(grep("EarlyStage",setPT$PTlabel)) + 1):(min(grep("MidStage", setPT$PTlabel))
 - 1)] <- "transition1_2"
 }

 if(min(grep("LateStage",setPT$PTlabel)) - max(grep("MidStage",setPT$PTlabel)) > 1){
        setPT$PTlabel[(max(grep("MidStage", setPT$PTlabel)) + 1):(min(grep("LateStage", setPT$PTlabel))
 - 1)] <- "transition2_3"
 }

 return(list(data,setGB,setPT,sel,gsData))

}

getTtest <- function(x) {

 refM <- x[rowMedians(x) >= min(colMedians(x,  keep.names=F)) & rowMedians(x) <=
 max(colMedians(x,  keep.names=F)), ]
 geneM <- x[!(rowMedians(x) >= min(colMedians(x,  keep.names=F)) & rowMedians(x) <=
 max(colMedians(x,  keep.names=F))), ]

 ttRes <- data.frame(gene=rownames(geneM), logFC=rep(1,nrow(geneM)), Pvalue=rep(1,nrow(geneM)),
 qval=rep(1,nrow(geneM)), lfdr=rep(1,nrow(geneM)))

 ref <- as.numeric(refM)

 for(i in 1:nrow(geneM)){

        gene <- as.numeric(geneM[i,])
        res <- t.test(ref,gene)
        ttRes[i,"Pvalue"] <- res$p.value
        ttRes[i,"logFC"] <- log2(mean(gene)/mean(ref))
```

```
        ttRes[i,"ensemblID"] <- strsplit(ttRes[i,"gene"],"\\|")[[1]][1]
        ttRes[i,"geneName"] <- strsplit(ttRes[i,"gene"],"\\|")[[1]][2]
        rm(res)

 }

 qobj <- qvalue(ttRes$Pvalue)
 ttRes$qval <- qobj$qvalues
 ttRes$lfdr <- qobj$lfdr

 ttRes <- merge(ttRes, ensembl2EG, by="ensemblID", all.x=T)
 ttRes <- ttRes[, c("gene","ensemblID","geneName","entrezID","logFC","Pvalue","qval","lfdr")]

 return(ttRes)

}

getENTREZG <- function(x) {

 ### genes <- getENTREZG(emlMx)

 genes <- data.frame(geneID=rownames(x), stringsAsFactors=F)

 for(i in 1:nrow(genes)){

        genes$ensemblID[i] <- strsplit(genes$geneID[i],"\\|")[[1]][1]
        genes$geneName[i] <- strsplit(genes$geneID[i],"\\|")[[1]][2]

 }

 genes <- merge(genes, ensembl2EG, by="ensemblID", all.x=T, all.y=F)
 rownames(genes) <- genes$geneID
 genes <- genes[rownames(emlM), ]

 rm(y)
 rm(yy)
 rm(ensembl2EG)
 rm(mapped_genes)

 return(genes)

}

getLimma <- function(x,z) {

 ### efit <- getLimma(emlM, emlPT)

 genes <- getENTREZG(x)
```

```
 y <- DGEList(counts=x, group=z$Stage, genes=genes)

 group <- y$samples$group
 design <- model.matrix(~0+group)
 colnames(design) <- sub("group", "", colnames(design))

 v <- voom(y,design,plot = FALSE)

 contr.matrix <- makeContrasts(
             MidvsEarly = Mid-Early,
             LatevsEarly = Late-Early,
             LatevsMid = Late-Mid,
             levels=v$design)

 vfit <- lmFit(v, design)
 vfit <- contrasts.fit(vfit, contrasts=contr.matrix)
 efit <- eBayes(vfit)

 rm(genes)
 rm(y)
 rm(design)
 rm(contr.matrix)
 rm(vfit)

 return(efit)

}

getGAGE <- function(x,y,z) {

 # x: limma output toptable
 # y: geneset
 # z: q.val cut-off to filter the GAGE results
 # test <- getGAGE(MidvsEarly,kegg.gs,0.1)
 # test <- getGAGE(MidvsEarly,reactome.gs,0.1)
 # test <- getGAGE(MidvsEarly,go.gs,0.1)

 x <- x[, c("entrezID","logFC")]
 x <- x[complete.cases(x), ]

 exp.fc <- x$logFC
 names(exp.fc) <- x$entrezID

 fc.kegg.p <- gage(exp.fc, gsets = y, ref = NULL, samp = NULL)

 pathUp <- data.frame(pathway=rownames(fc.kegg.p$greater), fc.kegg.p$greater, stringsAsFactors=F)
 pathUp <- pathUp[!is.na(pathUp$q.val) & pathUp$q.val < z, ]
```

```
 if(nrow(pathUp) > 0){pathUp$Effect <- "UP"}

 pathDown <- data.frame(pathway=rownames(fc.kegg.p$less), fc.kegg.p$less, stringsAsFactors=F)
 pathDown <- pathDown[!is.na(pathDown$q.val) & pathDown$q.val < z, ]
 if(nrow(pathDown) > 0){pathDown$Effect <- "DOWN"}

 if(nrow(pathUp) > 0 && nrow(pathDown) > 0){
         path <- rbind(pathUp,pathDown)
 }else if(nrow(pathUp) > 0){
         path <- pathUp
 }else if(nrow(pathDown) > 0){
         path <- pathDown
 }

 path <- path[, c("pathway", "Effect", "q.val", "p.geomean", "stat.mean", "p.val", "set.size", "exp1")]
 path <- path[order(path$q.val,path$Effect), ]
 rownames(path) <- 1:nrow(path)

 rm(fc.kegg.p)
 rm(pathUp)
 rm(pathDown)
 rm(exp.fc)

 if(nrow(path) > 0){return(path)}else{return(NULL)}

}

###
### test-run
### > getwd()
### [1] "/media/yc790/Samsung_T55/work/evans/NCICCR-DLBCL"
### For each RNASeq data set, we have DGE and GSEA/pathway analysis results from Mid_vs_Early,
 Late_vs_Mid, Late_vs_Early, withinEarlyOnly, withinMidOnly,
### and withinLateOnly. For each GSEA/pathway analysis, we run for reactome, TFT, kegg, and go
 individually
###

library(limma)
library(edgeR)
library(gage)
library(robustbase)
library(qvalue)
options(digits=2)

# to read in original counts table as a matrix
x <- readRDS("testData/NCICCR-DLBCL_htseq_counts.rds")

# to read in entrez id to gene symbol mapping
```

```
glen <- readRDS("testData/ensembl_ext_gene_name_mean_tx_length.rds")
ensembl2EG <- readRDS("testData/org.Hs.egENSEMBL.ensembl2EG.rds")

# to do TMM normalization and return log2 CPM as a matrix
# Warning message: In filterByExpr.DGEList(y) :All samples appear to belong to the same group.
# It's OK because no sample groups were defined so far.
# If the input is already normalized, using lcpm <- getLCPM(x,glen,TRUE)
# the normalized data should be a numeric matrix and log2 transformed
lcpm <- getLCPM(x,glen,FALSE)

# to do data transformation and return a list with 5 elements
gbpt_lcpm <- getGB_PT(lcpm)

# reordered lcpm matrix
data <- gbpt_lcpm[[1]]

# GB order of genes as a numeric vector
setGB <- gbpt_lcpm[[2]]

# PT order of patients/sample as a data frame
# setPT$sample: sample name
# setPT$PT: calculated PT value
# setPT$PTlabel: assigned stage for each sample
 "EarlyStage","transition1_2","MidStage","transition2_3","LateStage"
setPT <- gbpt_lcpm[[3]]

# calculated number of samples in EarlyStage/MidStage/LateStage
sel <- gbpt_lcpm[[4]]

# the transformed final data as a matrix
gsData <- gbpt_lcpm[[5]]

### to save the data as data frame for clustering using other tools please uncomment the following line
### the data will be saved in sub-difectory testResult asa gsData.txt
# write.table(data.frame(geneID=rownames(gsData), gsData), file="testResult/gsData.txt", sep="\t",
 row.names=F)

# to calculate median-shift values for EarlyStage/MidStage/LateStage sample matrixes
esData <- getMS(gsData[, setPT[setPT$PTlabel == "EarlyStage", "sample"]])
msData <- getMS(gsData[, setPT[setPT$PTlabel == "MidStage", "sample"]])
lsData <- getMS(gsData[, setPT[setPT$PTlabel == "LateStage", "sample"]])

# to do DGE within EarlyStage/MidStage/LateStage respectively by T test and qvalue
# For each analysis the return is a data frame, for example, for EarlyStage result res_esData
# res_esData$gene : chr "ENSG00000063322|MED29" "ENSG00000176396|EID2"
 "ENSG00000105197|TIMM50" "ENSG00000110442|COMMD9" ...
# res_esData$ensemblID
# res_esData$geneName
```

```
# res_esData$entrezID
# res_esData$logFC
# res_esData$Pvalue
# res_esData$qval
# res_esData$lfdr
res_esData <- getTtest(esData)
res_msData <- getTtest(msData)
res_lsData <- getTtest(lsData)

### to save the result please uncomment the following 3 lines
# write.table(res_esData, file="testResult/DEG_EarlyStage.txt", sep="\t", row.names=F, quote=F)
# write.table(res_msData, file="testResult/DEG_MidStage.txt", sep="\t", row.names=F, quote=F)
# write.table(res_msData, file="testResult/DEG_LateStage.txt", sep="\t", row.names=F, quote=F)


# to do DGE for MidvsEarly, LatevsEarly, LatevsMid respectively using limma/edgeR

emlPT <- setPT[grep("Stage", setPT$PTlabel), ]
emlM <- 2^data[, emlPT$sample]
colnames(emlPT)[3] <- "Stage"
emlPT$Stage <- gsub("Stage","",emlPT$Stage)
emlPT$Stage <- factor(emlPT$Stage, levels=c("Early","Mid","Late"))

efit <- getLimma(emlM, emlPT)

MidvsEarly <- topTable(efit, coef = "MidvsEarly", n = Inf, sort = "B")
LatevsEarly <- topTable(efit, coef = "LatevsEarly", n = Inf, sort = "B")
LatevsMid <- topTable(efit, coef = "LatevsMid", n = Inf, sort = "B")

### to save the result and volcano plots please uncomment the following lines
# write.table(MidvsEarly, file="testResult/toptable_MidvsEarly.txt", sep="\t", row.names=F, quote=F)
# write.table(LatevsEarly, file="testResult/toptable_LatevsEarly.txt", sep="\t", row.names=F, quote=F)
# write.table(LatevsMid, file="testResult/toptable_LatevsMid.txt", sep="\t", row.names=F, quote=F)
# saveRDS(MidvsEarly, file="testResult/optable_MidvsEarly.rds")
# saveRDS(LatevsEarly, file="testResult/toptable_LatevsEarly.rds")
# saveRDS(LatevsMid, file="testResult/toptable_LatevsMid.rds")

# png(file="testResult/volcanoplot_MidvsEarly_B_value.png", width = 900, height = 720, units = "px",
 pointsize = 16)
# volcanoplot(efit, coef="MidvsEarly", style = "B-statistic", highlight=10, names=efit$genes$geneName,
 hl.col="red")
# dev.off()

# png(file="testResult/volcanoplot_LatevsEarly_B_value.png", width = 900, height = 720, units = "px",
 pointsize = 16)
# volcanoplot(efit, coef="LatevsEarly", style = "B-statistic", highlight=10, names=efit$genes$geneName,
 hl.col="red")
# dev.off()
```

```
# png(file="testResult/volcanoplot_LatevsMid_B_value.png", width = 900, height = 720, units = "px",
 pointsize = 16)
# volcanoplot(efit, coef="LatevsMid", style = "B-statistic", highlight=10, names=efit$genes$geneName,
 hl.col="red")
# dev.off()

kegg.gs <- readRDS("testData/kegg_hsa_gs.rds")
reactome.gs <- readRDS("testData/MSigDB_reactome_gs.rds")
go.gs <- readRDS("testData/MSigDB_GO_gs.rds")
tft.gs <- readRDS("testData/MSigDB_tft_gs.rds")

for(i in c("MidvsEarly","LatevsEarly","LatevsMid","res_esData","res_msData","res_lsData")){

 for(j in c("kegg","reactome","go","tft")){

        cat(paste(i,j,"\n", sep="\t"))

        test <- getGAGE(eval(parse(text = i)),eval(parse(text = paste(j,"gs",sep="."))),0.1)

        ### to save each test result please unment the following line to save results as text files
        ### in sub directory testResult
        # write.table(test,file=paste("testResult/GAGE",i,j,"UpDown.txt",sep="_"),sep="\t", row.names=F)

 }

}

### End of code
```
