# Supplementary material for: An Analysis of Transcriptomic Burden Identifies Biological Progression Roadmaps for Hematological Malignancies and Solid Tumors
Source: Biomedicines. 2022 Oct 27;10(11):2720. doi: 10.3390/biomedicines10112720 (PMC9687799; doi:10.3390/biomedicines10112720)
Supplement: Supplementary file 1 [file biomedicines-10-02720-s001.zip › Supplemental Figures S1-8.pdf]

Supplemental Figure S1

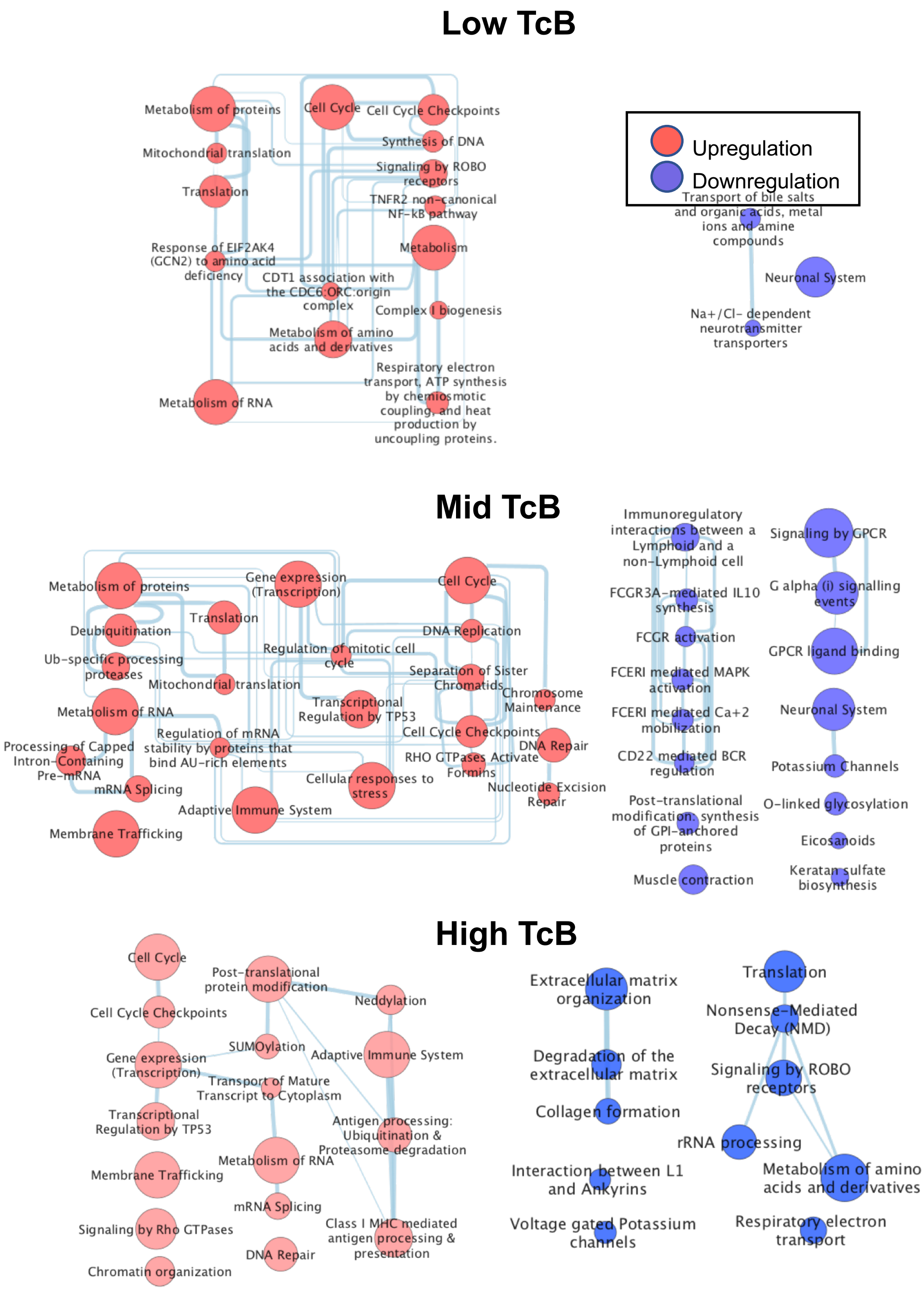

Figure S1. Biological pathway networks from enrichment analysis comparing up and down regulated against each TcB group medians of Cornell DLBCL

Supplemental Figure S2

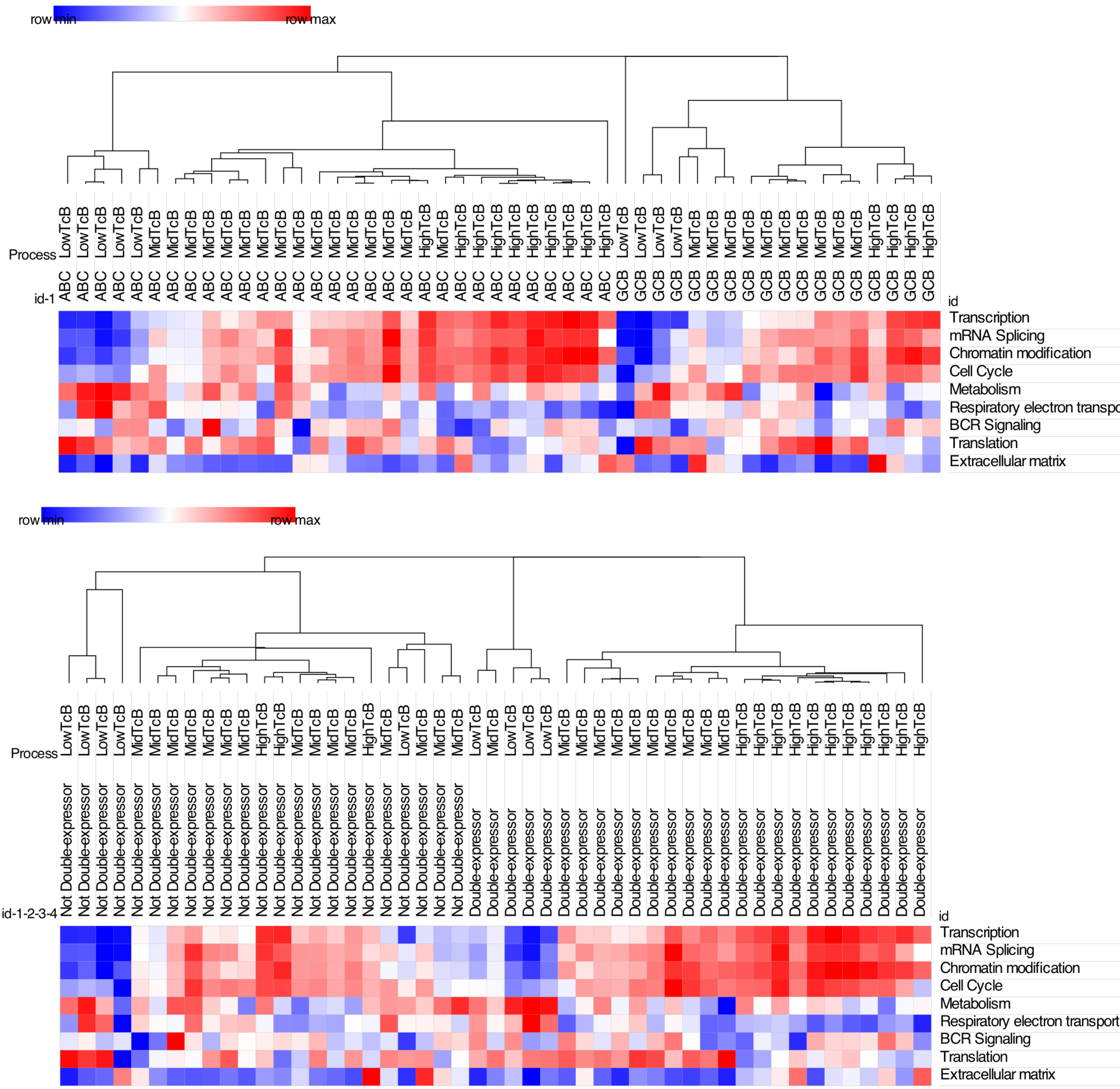

Figure S2. Pathway analysis of Cornell DLBCL training set sorted by TcB. Heatmap representation indicate higher order biological functions enriched by TcB are independent of lymphoma cell of origin (Germinal Center B cells (GCB) and Activated B Cells) (TOP PANEL) and molecular subtypes (MYC and BCL2 double expresser) (BOTTOM PANEL).

Supplemental Figure S3A

DLBCL Cornell

Hierarchical

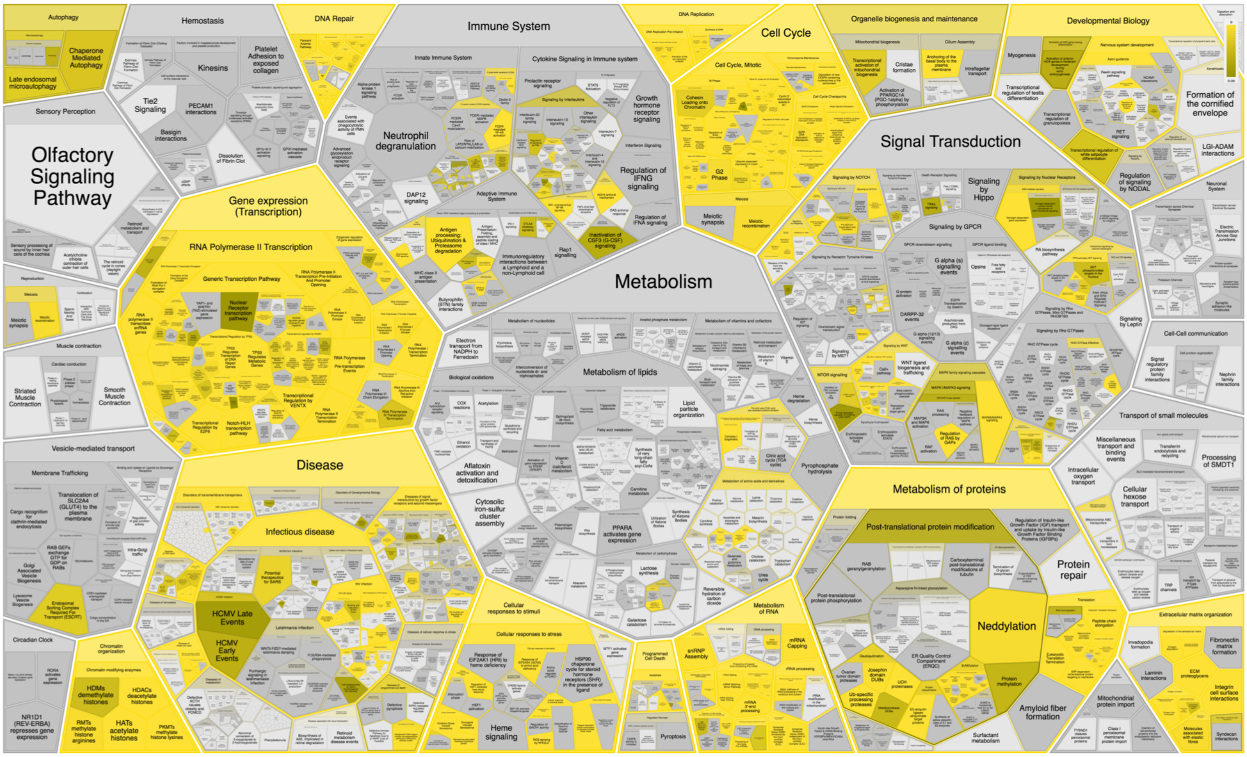

Collapsed by Higher Order

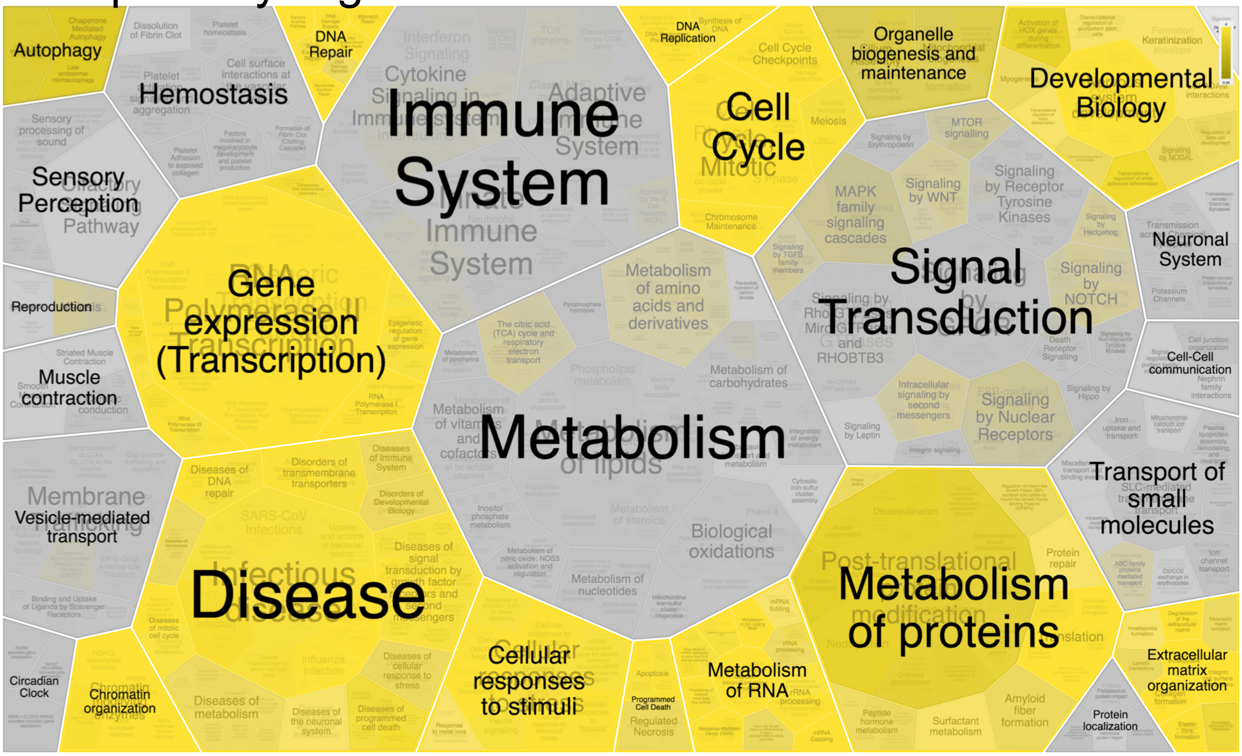

Figure S3 (A-F). Pathway analysis of TcB enriched genesets from hematological malignancies and solid tumor. Reactome foam representation of enriched biological processes identified using significant gene sets determined from all TcB groups, shown as detailed processes (TOP PANEL) or flattened by higher-order process (BOTTOM PANEL).

Supplemental Figure S3B

DLBCL NCI

Hierarchical

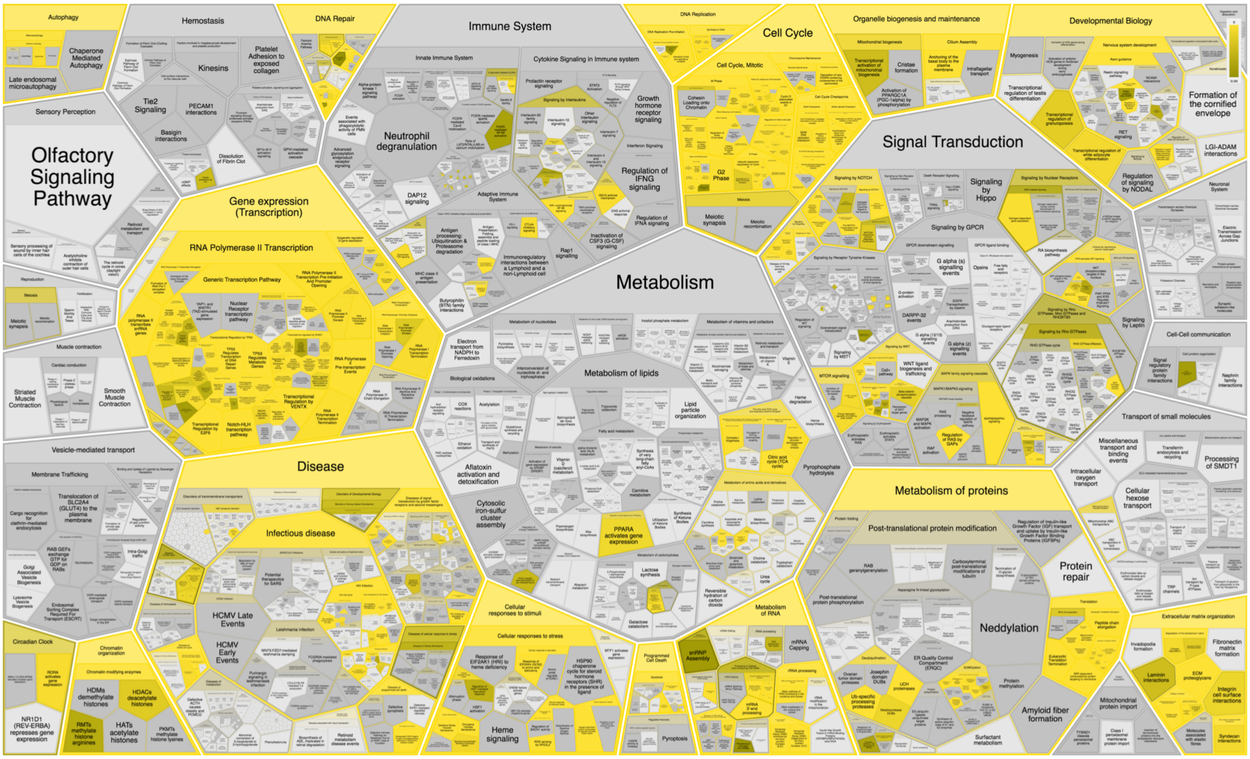

Collapsed by Higher Order

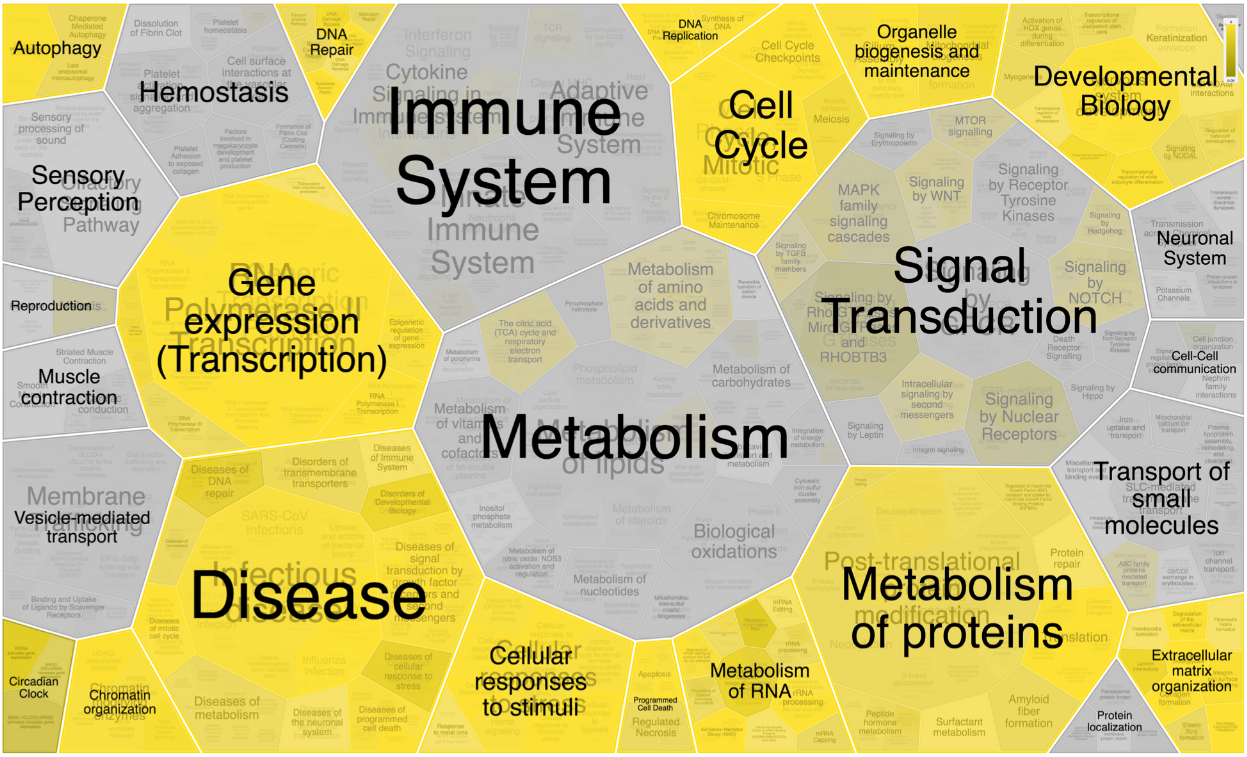

Supplemental Figure S3C

TARGET ALL

Hierarchical

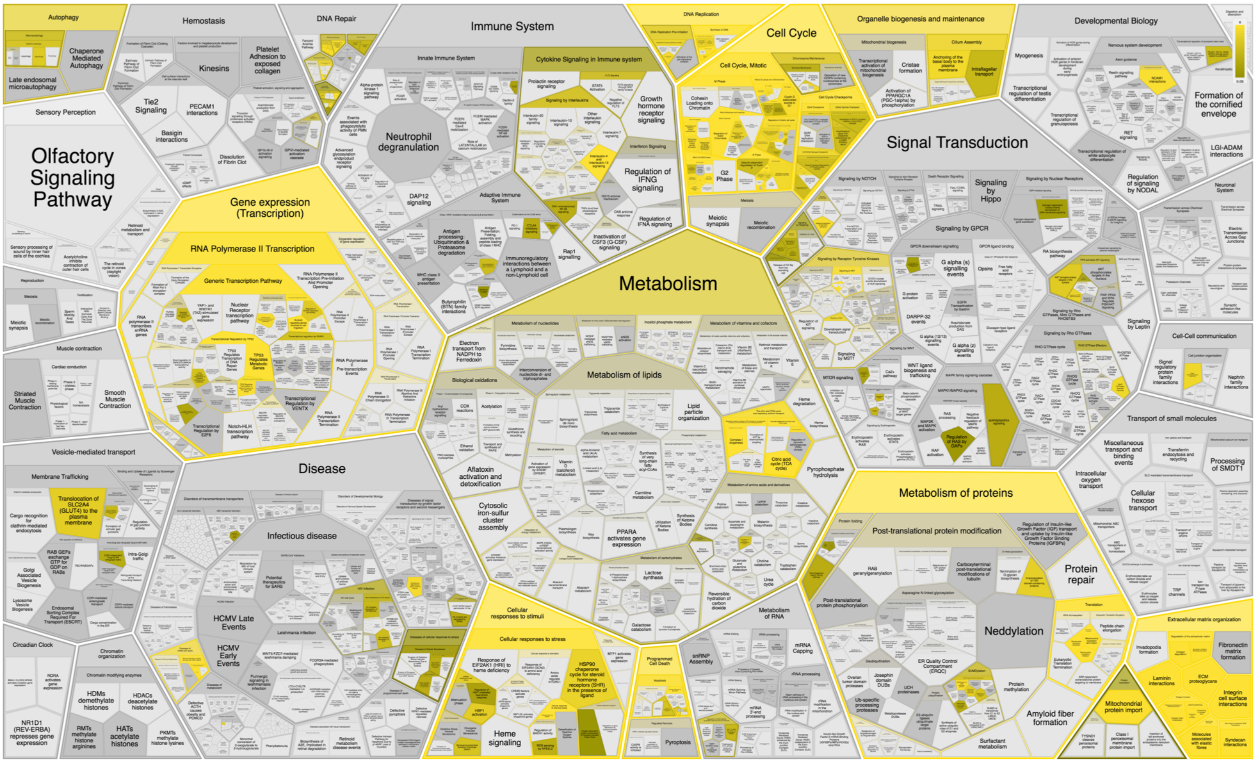

Collapsed by Higher Order

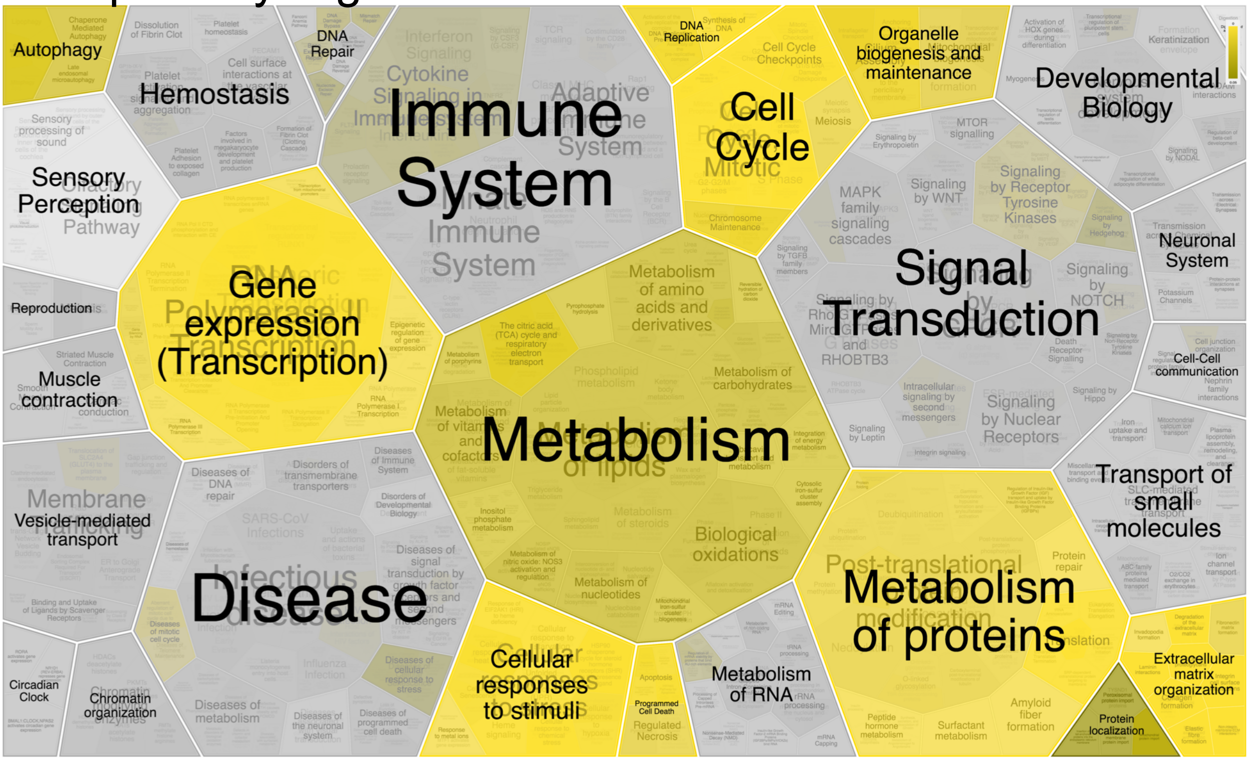

Supplemental Figure S3D

AML

Hierarchical

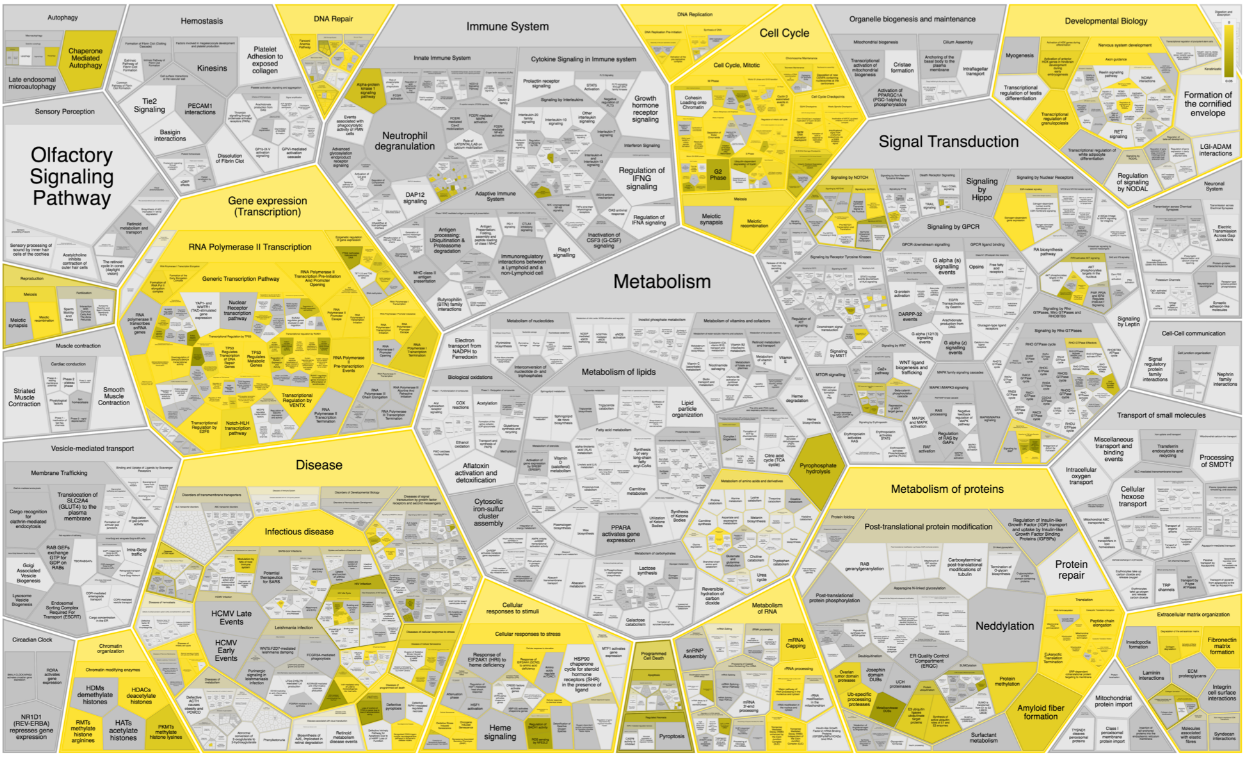

Collapsed by Higher Order

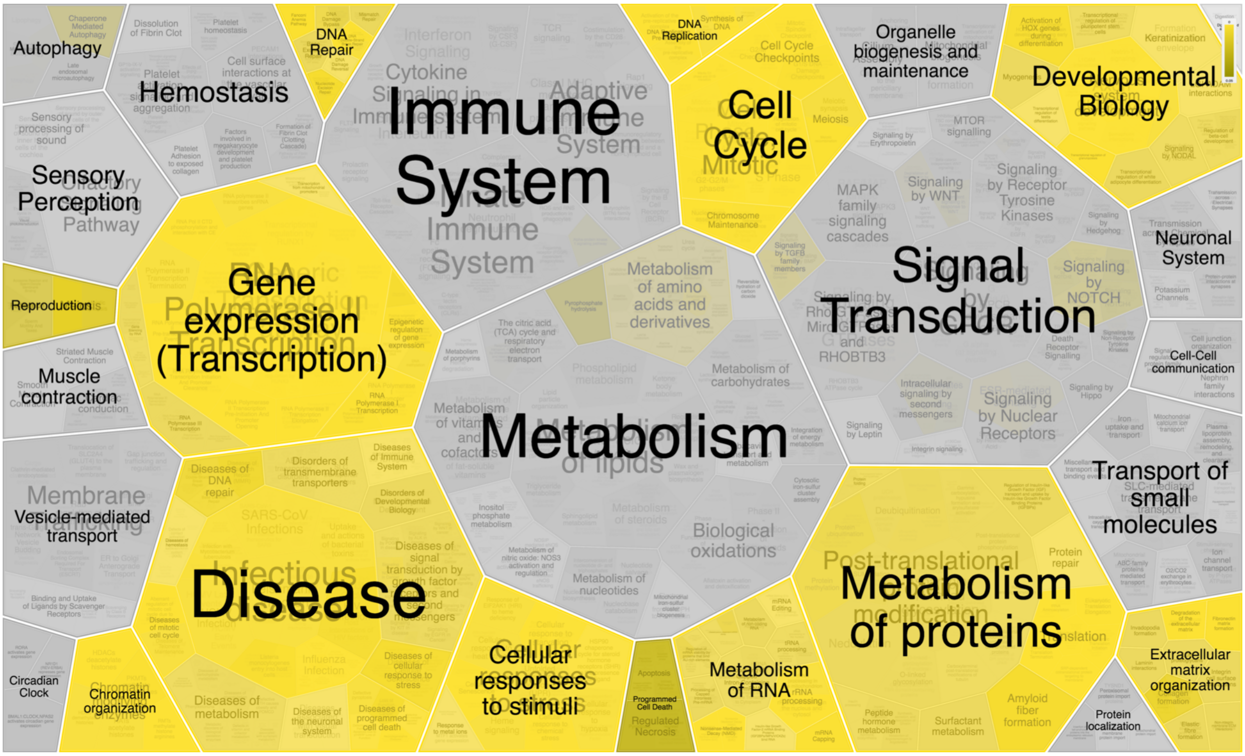

Supplemental Figure S3E

Breast cancer

Hierarchical

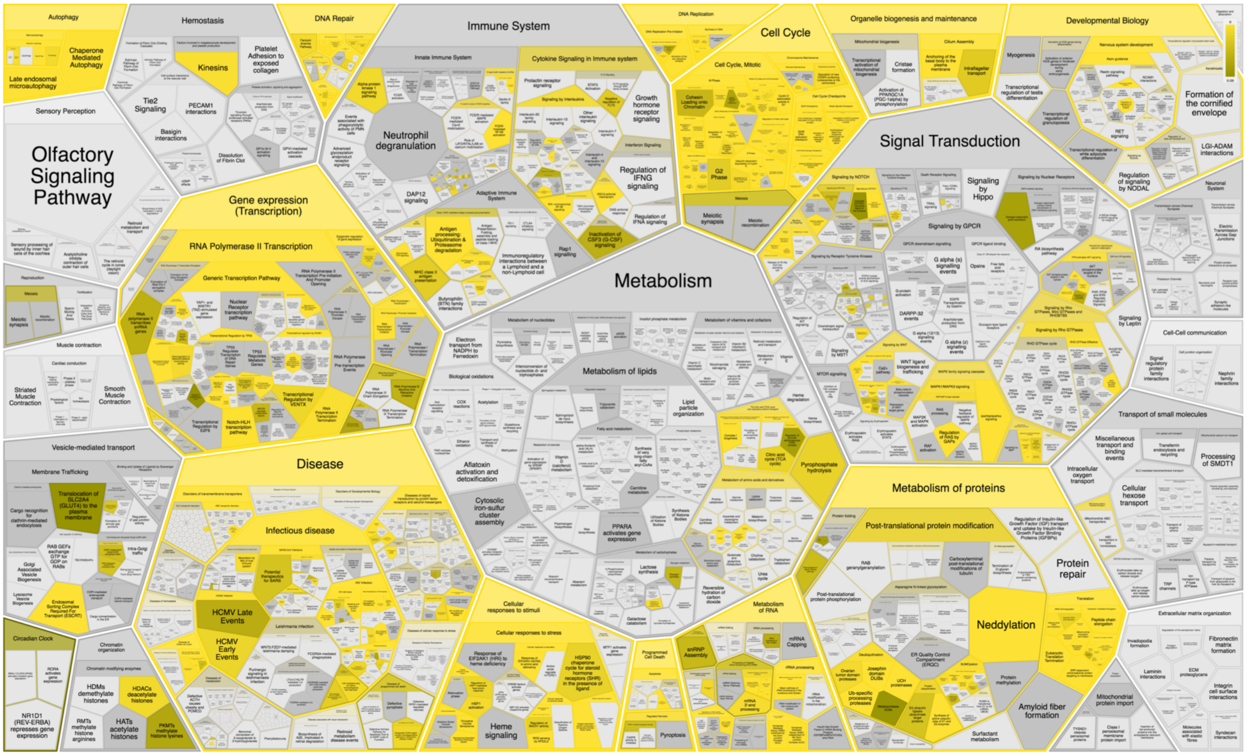

Collapsed by Higher Order

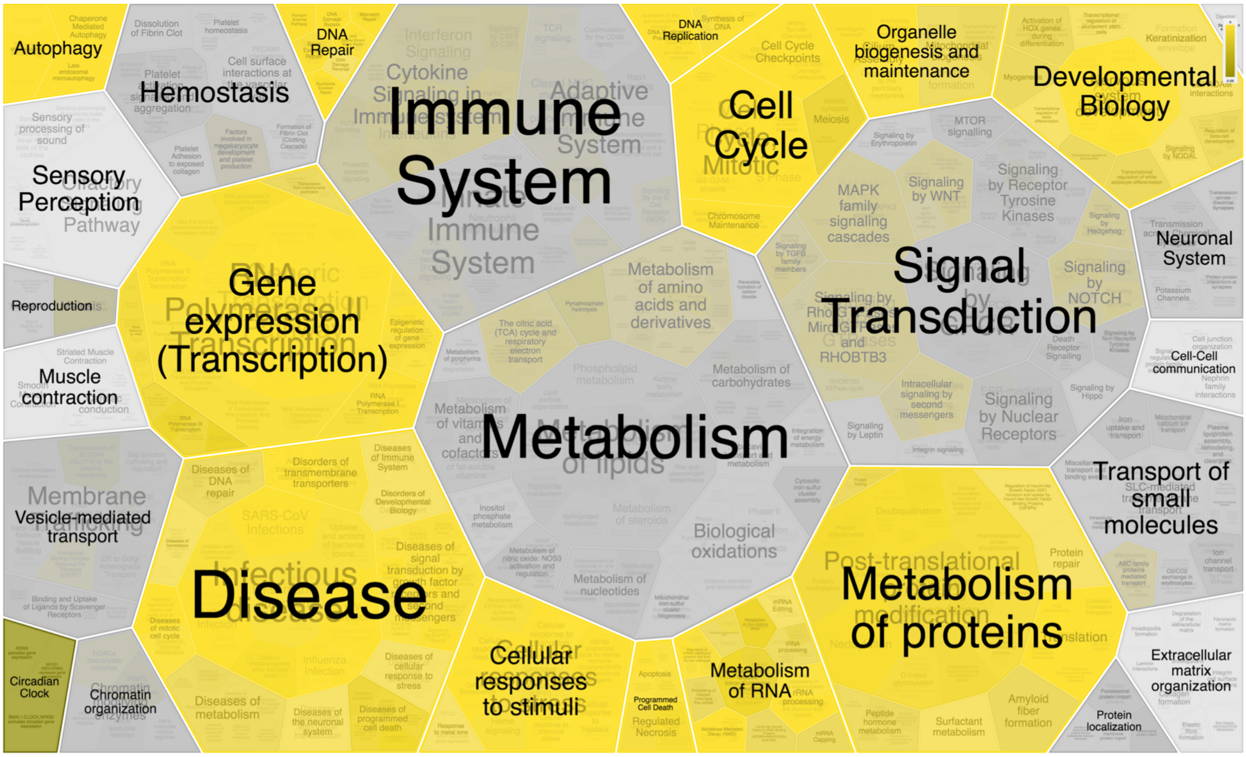

Supplemental Figure S3F

Bladder cancer

Hierarchical

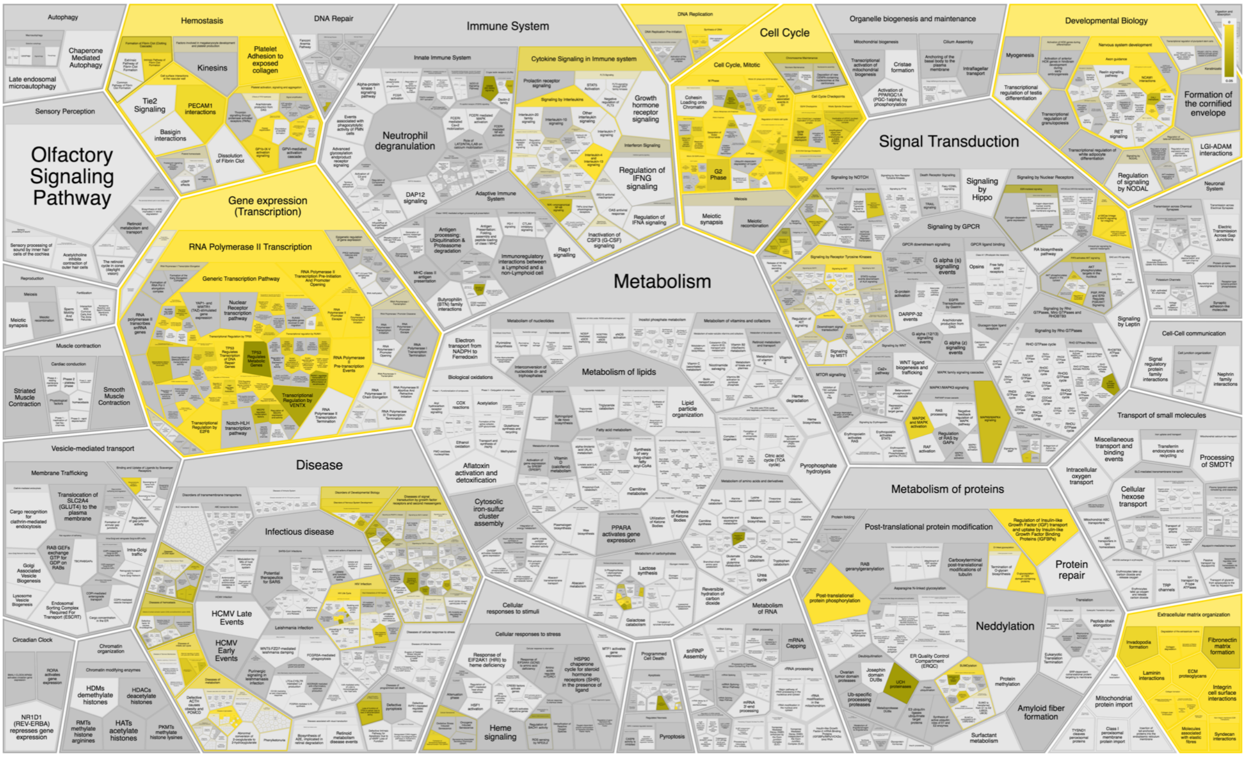

Collapsed by Higher Order

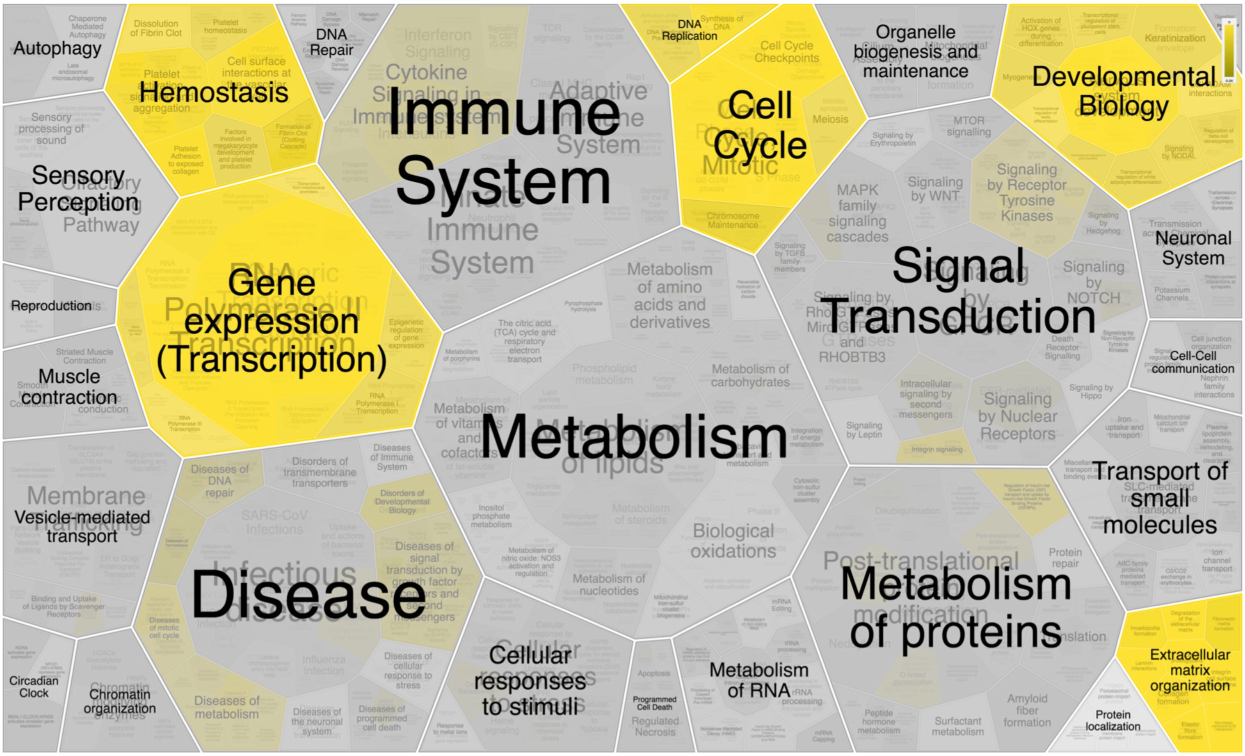

Supplemental Figure S4

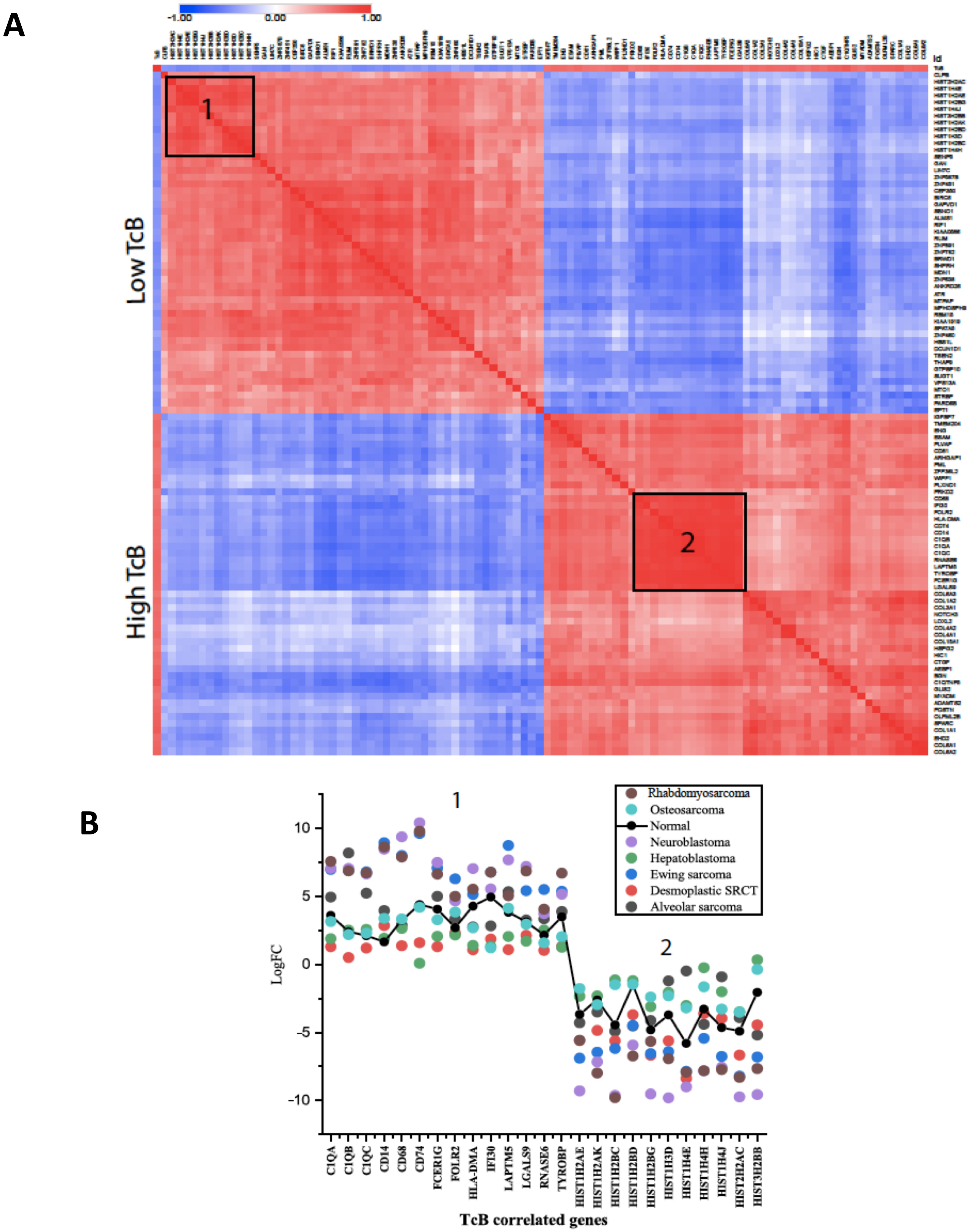

Figure S4. A) Heatmap representation from TcB –gene correlation analysis from pan pediatric extracranial solid tumor panel show genes that are highly correlated by low or highTcB. B) Scatter plot of gene represented in clusters 1 & 2 marked in A, show average log fold change tumor vs normal tissues.

Supplemental Figure S5

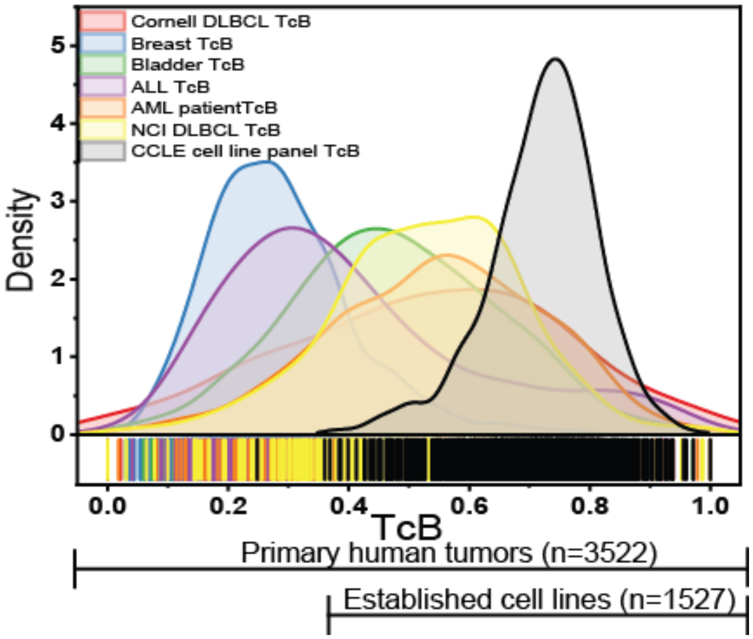

Plot of kernel density shows equal distribution of TcB values (x-axis) across 6 tumor datasets (n=3522) and skewed distribution in CCLE cell line panel (n=1527).

Supplemental Figure S6

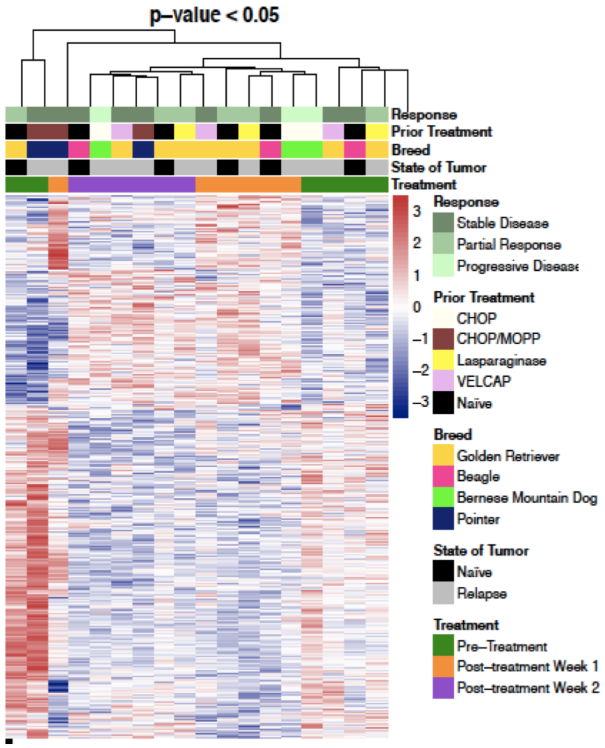

Figure S6: Canine lymphoma BKM120 trial and gene expression analysis. A) Heatmap represents overall changes in significant gene expression patterns across canine lymphoma specimens obtained prior to therapy and one and three weeks post therapy. B) Network representation from geneset enrichment analysis using reactome database show up or down regulated processes and interactions from overall treatment among canine lymphomas.

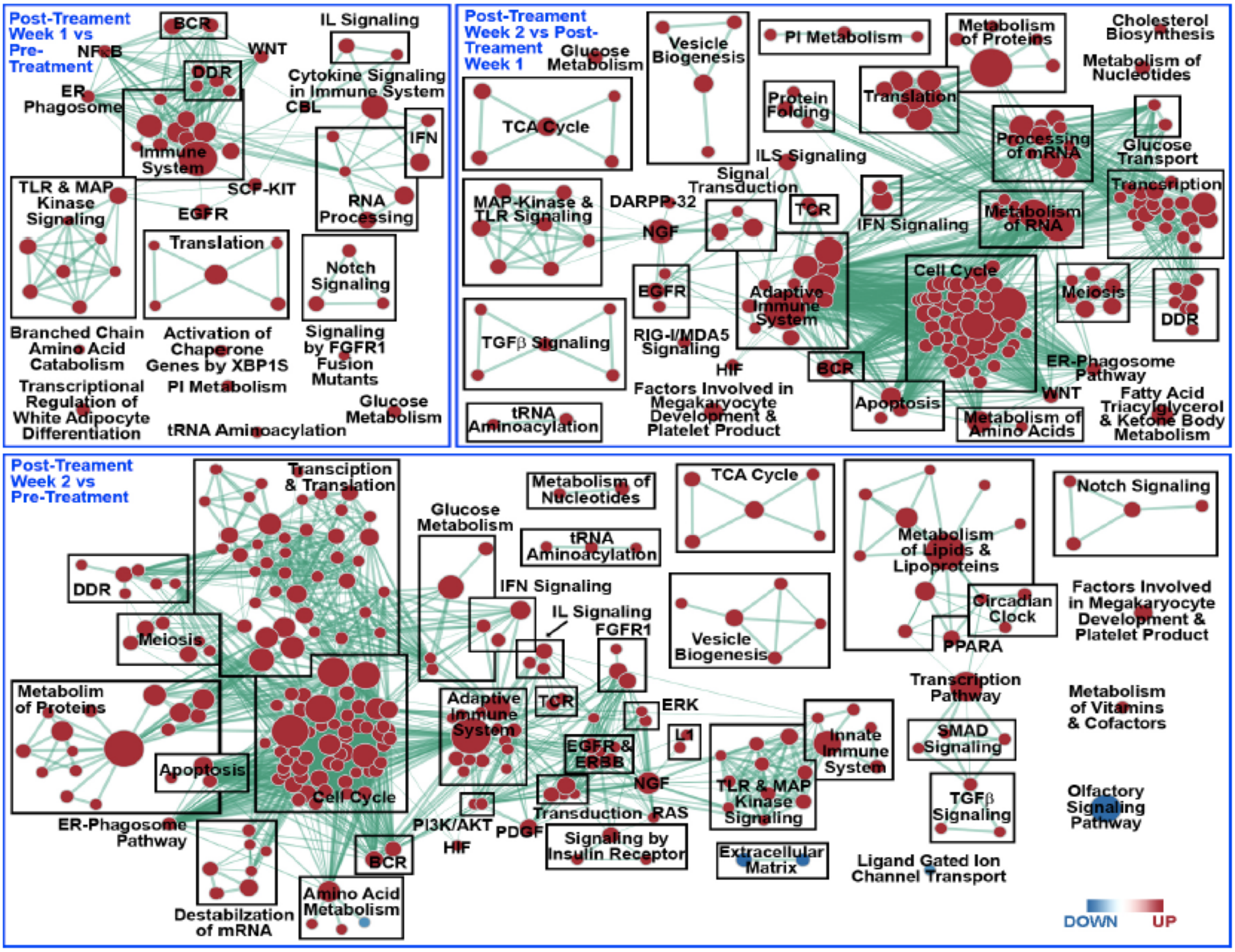

Supplemental Figure S7

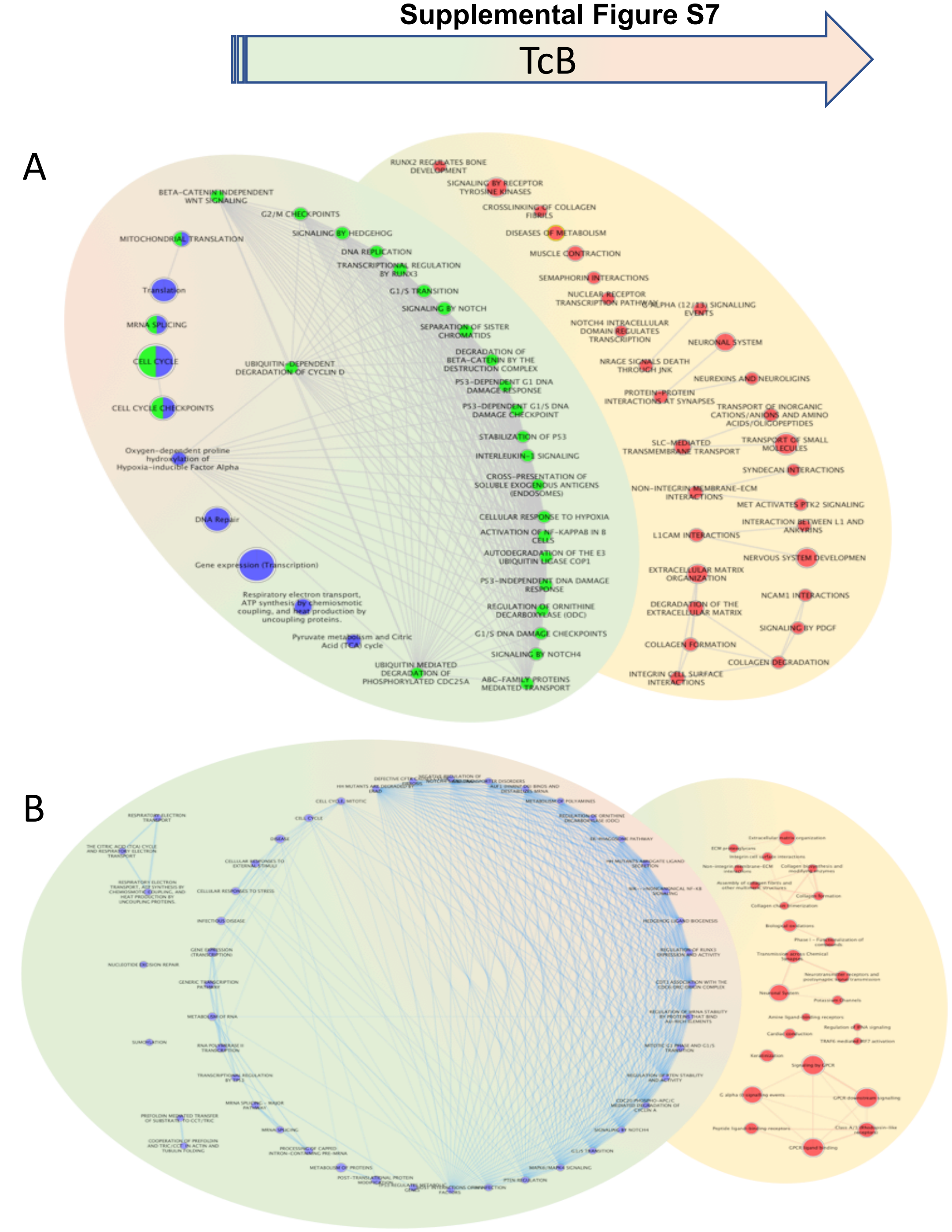

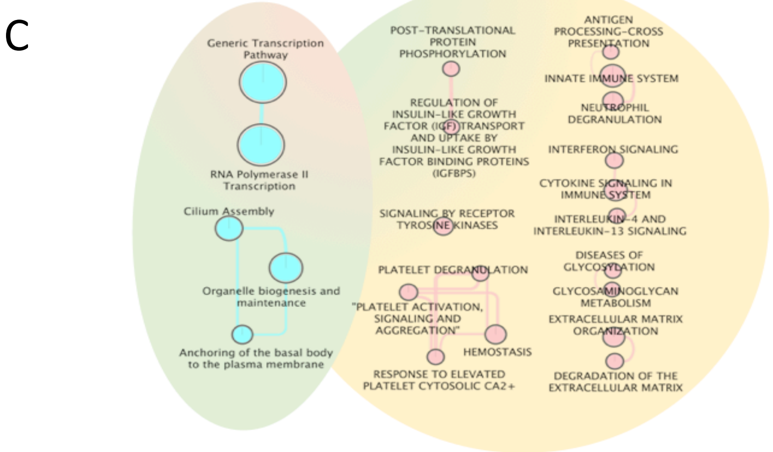

Figure S7. Biological progression network in A) DLBCL, B)ALL and C) Pediatric solid tumors. Cytoscape representation of biological progression networks enriched from TcB analysis, with nodes representing biological process from lowTcB (blue), midTcB (green) and highTcB (red), with edges indicating interactions among enriched biological process.

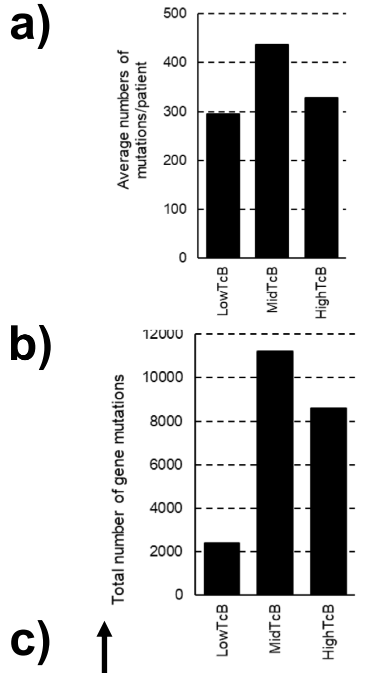

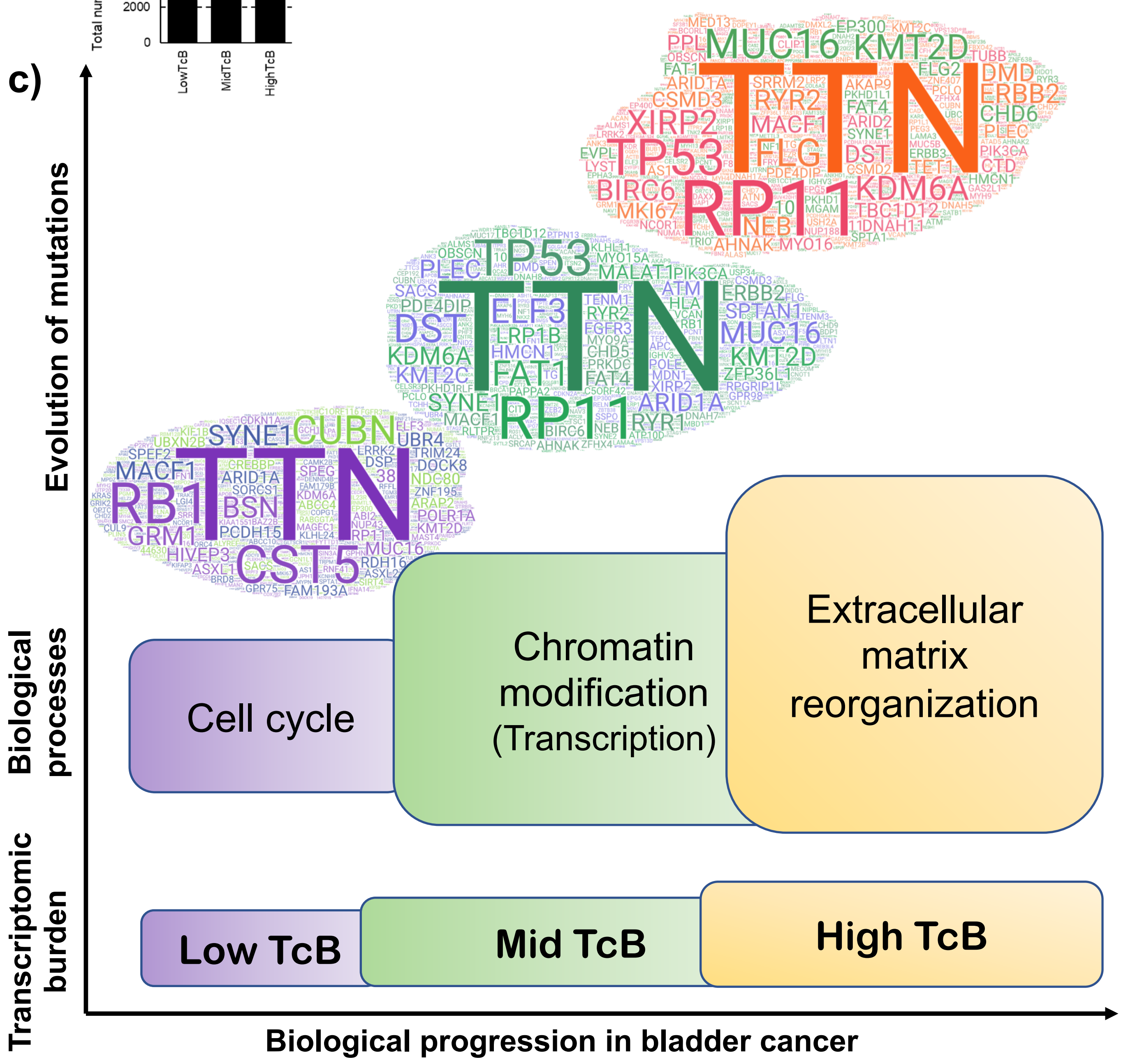

Figure S8. Gene alterations and TcB trajectory. Gene alterations associated with each bladder cancer patient's transcriptome were aligned by TcB for projection of gene alteration evolution. Graphs show (A) average number of genes altered per patient; and (B) total number of gene alterations across all TcB subgroups. C) Word cloud analysis for genes frequently altered, together with progression of higher order biological processes associated with increasing TcB trajectory.
